# “I don’t want to go anywhere”: A qualitative study with patients with Graves’ Disease and Ophthalmopathy interviewed at a public university specialized outpatient service in Brazil

**DOI:** 10.1192/j.eurpsy.2023.1617

**Published:** 2023-07-19

**Authors:** E. R. Turato, L. S. Valladão, D. E. Zantut-Wittmann, C. F. Casagrande, F. S. Santos

**Affiliations:** Laboratory of Clinical-Qualitative Research, State University of Campinas, Campinas, Brazil

## Abstract

**Introduction:**

Knowing what “kind of patient” search the doctor is relevant to handle his/her treatment and care. What emotional meanings do people attach to their diagnosis, therapy, and care in general? Graves’ Disease has an impact on the metabolism, which directly affects behavior. Patients can be affected by Graves’ Ophthalmopathy at any stage of the disease. This usually leads to greater changes in facial physiognomy which may or may not be accompanied by typical symptoms of hyperthyroidism. Changes in appearance and visual symptoms often lead to a rapid search for treatment. Understanding the symbolic aspects of the condition can help clinicians to manage emotionally, leading to substantial improvements such as the adhesion to the treatment.

**Objectives:**

To understand and interpret psychodynamically the perceptions and emotional meanings related to Graves’ Disease with Ophthalmopathy and hyperthyroidism as reported by patients at an endocrinology-specialized outpatient service.

**Methods:**

Clinical-qualitative design of Turato. Data is collected using Semi-directed interviews with open-ended questions in-depth carried out with patients at an specialized outpatient service. Interview material was audio-recorded and fully transcribed. The material was treated by Clinical-Qualitative Content Analysis of Faria-Schützer. It is based on psychodynamic concepts from the Medical Psychology theoretical framework, and which main author is Michael Balint. The sample will be closed by the Theoretical Saturation of Information criterion according Fontanella. The interviewer was a male psychologist and doctoral student in the Health Mental Area. The finding validation has occurred by peers at the Laboratory of Clinical-Qualitative Research, State University of Campinas.

**Results:**

This presentation refers to partial findings from a sample of a total of three participants as part of the doctoral project by the first author. The analysis of data collected so far indicates three designed categories of analysis: 1) “I wasn’t like that before”: impacts on physiognomy - the perception of social stigma and the difficulty of managing the illness. 2) “This disease is a mess”: the symptoms affect all aspects of the patient’s life. 3) Problematic emotions management: double anxiety in hyperthyroidism - hormonally based anxiety and psychological anxiety related to the impact of the medical diagnosis.

**Conclusions:**

The findings shall aid medical personnel in better grasping patients’ emotional perceptions of their illness and medical care. The level of communication between the professional and the patient can be improved by involving the patient as an active protagonist in the well-being process. This could also lead to more effective management and greater adherence to the therapeutic process.

**Disclosure of Interest:**

None Declared

